# A Case of Typhoid Pneumonia Cured with Hyos.^DMM^

**Published:** 1893-02

**Authors:** Samuel A. Kimball

**Affiliations:** Boston, Mass.


### A CASE OF TYPHOID PNEUMONIA CURED WITH HYOS.$^{DMM}$.

Samuel A. Kimball, M. D., Boston, Mass.

Miss ——, a delicate child seven years of age, has never been strong since she was vaccinated when nine months old. Until then she was a plump, healthy baby, but after the vaccination she became thin, was constantly ailing, and whenever sick was very ill, having had the measles, whooping cough, and scarlet fever in a most aggravated form.

I was called to see her in the evening of March 11th, 1891. She then had been ill about five days with pneumonia, under the care of a so-called homœopathic physician, taking from three to five remedies daily, and at the time of my visit Iodine and Bromium in hourly alternation. She was much emaciated, lying on her back in a half sleeping, delirious condition, with moaning, crying out, jumping and starting.

Sleeping with half open eyes.

Moaning, crying out and screaming during sleep. Irritable on waking. Nostrils dilate during respiration.

Tongue dry, brown; lips and teeth covered with sordes.

Urine scanty, passed once daily; involuntary urine this A. M., staining reddish brown. Hard, dry cough with moaning before coughing, much rattling in the chest, but rarely any expectoration. There was dullness on percussion, with moist râles, over the whole right chest, front and back. Temperature, 103.5° ; pulse, 120 ; respiration, 50 to 60.

The case was carefully studied. Lyc. and Hyos. were each more prominent than other remedies, but on account of the irritability on waking, the dilatation of the nostrils during respiration, the reddish stain from the involuntary urine and the attack being right sided, Lyc. was selected and two doses of the DMM potency, Swan, were given in water three hours apart.

March 12th, 8 A. M.—She slept fairly well, but would moan, cry out and start up in sleep before coughing. This morning she is in a semi-delirious condition most of the time, restless and irritable, with moaning and crying out. Has had hands on the genitals. Cough the same, physical signs the same. Tongue dry, brown ; sordes still on the teeth. Nose obstructed. No urine since yesterday noon. Temperature, 102° ; pulse, 120 ; respiration, 48 to 60.

At 12.30 P. M. temperature, 103.4°. Passed considerable urine at 12.45 P. M., after two or three painful, ineffectual efforts. Involuntary urine at 1.30 P. M., with slight yellow stool that stained the sheet an orange yellow. She coughs and swallows the expectoration. At 1.45 P. M. another dose of Lyc.DMM, in water, was given. Temperature at 3 P. M., 102° ; respiration, 48 to 60. Yellow stool at 4 P. M., not watery. Desire for light, wishes the curtain pulled up.

Feet cold this afternoon. Answers correctly when spoken to, but immediately becomes delirious or falls asleep.

During the evening she moaned and talked in sleep, called for her papa, seemed be in fear, afraid of bears and of falling. About 10 P. M. as her mother approached the bed, she started up with staring eyes, widely dilated pupils, shouted at and struck her mother, as if in great fear. She sprang at me in the same manner and seemed terrified, as if we were dreadful objects that she was trying to frighten away.

The mental symptoms were now more pronounced than at any time.

The involuntary stool and urine in the morning, the character of the delirium during the day, answering correctly when spoken to but immediately relapsing into delirium, the terror at night with staring eyes and widely-dilated pupils, all pointed strongly to Hyos. Since the Lyc, there had been no particular improvement, not as much as one would expect from the simillimum, and the mental condition was worse.

Hyos.$^{DMM}$, Swan, was given in water at 10 P. M., and another dose in three hours.

March 13th, 8 A. M.—She had a restless night with more talking than moaning, imagined herself lost. Restless with tossing about before coughing.

A small stool before midnight.

The latter part of the night slept with eyes closed, no more fear.

This morning she is rational, mind quite clear, but she is very weak. Tongue dry, lips dry.

Temperature, 101.4° ; pulse, 144 ; respiration better.

During these few days it was very difficult to nourish her. All she would take was two or three teaspoonfuls of cream and water alternating with the same amount of unfermented grape juice every half-hour, and at times even this was refused.

5 P. M.—Has had a quiet and comfortable day and has taken food well.

No delirium. A soft, yellow stool at 2.30 P. M. Fine râles all over the right chest, and less dullness in the upper part, resolution was beginning from above downward. Tongue moist. Temperature, 102.4°.

March 14th.—Had a good night, passed urine in the night, is perfectly rational, eats well, tongue moist. Temperature, 100.4°.

From this time her improvement was rapid. The cough continued hard and dry with pain in the right chest.

In about a week, improvement ceasing, she received a dose dry of Sulph.$^{cm}$, Johnstone, followed by complete recovery, and since then her general health has been better than ever before.

### Discussion.

Dr. Campbell—I noticed that when Dr. Baylies delayed the giving of the medicine, the improvement was more marked, and when he ceased altogether it was still more marked and convalescence occurred sooner.

Dr. Long—I take exception to a point in the treatment of both cases, not so marked in Dr. Kimball's case as in Dr. Baylies'—that is, the frequent use of the thermometer.

Dr. Baylies—The temperature was taken by a trained nurse, before my arrival, and its continuance allowed without injury to the patient.

Dr. Long—Who runs the case; the trained nurse or the doctor? Would you allow a trained nurse to take the temperature every few minutes in typhoid?

Dr. Farley—Do you think this is another poultice?

Dr. McLaren—What is the objection to the use of the thermometer? I should like to know whether it is the same as mine.

Dr. Long—My objection is: first, it is apt to make the physician uncertain and nervous, and to interfere with his sound judgment in the selection of a remedy; second, it is very apt to cause disturbance with the patient and the friends. In other words, I think it causes unnecessary work and trouble on the part of the patient and his friends.

Dr. McLaren—I can give an instance of the mongrel use of the thermometer in the case of an old gentleman who was in a bad way. It became necessary, the friends thought, to hold a consultation with a mongrel. He came in with a thermometer, put it under the arm and also under the tongue, shook his head and passed it around. When he got down-stairs he said that the man was going to die in a week. He thought no treatment was of any use. He based his judgment on the thermometer alone. The case, however, lived six weeks on a CM potency; the DM held him two weeks more. He lived, altogether, two months after the consultation.

Dr. S. Close—Dr. Wells used to tell a story illustrating the abuse of the thermometer. He was called to see a young medical student, who imagined he had a fever, and had been taking his own temperature very frequently. He informed Dr. Wells of the daily run of his temperature. The doctor took the thermometer away from the patient, told him not to worry about the fever, and the next day he was well.

Dr. Baylies—This use of the thermometer was, for me, almost unprecedented. I don't suppose I have used it ten times in my life. I regard it as furnishing no indication for homœopathic treatment. The nurse took the temperature, and as I found no bad results I allowed her to continue.

Dr. Kimball—The thermometer had not the slightest signficance to me in the selection of the remedy. I do not often use the thermometer in my practice, but it sometimes gives us important information.

Dr. W. L. Morgan—I was called to see a case that was given up as hopeless, two weeks ago. I found the patient very cold, temperature $96\frac{7}{8}°$, but a single dose of Carbo-veg.$^{cm}$ raised the temperature to normal, and the patient was doing well when I left home.

Dr. Sawyer—I think there are two sides to this thermometer question. When the grippe was prevalent in the West we had some peculiar fever symptoms which one would be apt to miss without a thermometer. The temperature ran very low when it was not indicated by the pulse nor by feeling of the body. These cases under old-school treatment, which was usually successive doses of Quinine, died every time. The same kind of cases under one dose of Quinine high recovered every time.

